# Free Gingival Graft for External Cervical Root Resorption in a Non‐Esthetic Mandibular Premolar: A Case Report

**DOI:** 10.1155/crid/8865188

**Published:** 2026-02-09

**Authors:** Lucrezia Parma-Benfenati

**Affiliations:** ^1^ Department of Periodontics and Oral Medicine, UMICH, University of Michigan, Ann Arbor, USA, umich.edu

**Keywords:** case reports, composite resins, dental restoration, free gingival graft, gingival recession, keratinized gingiva, MeSH, periodontal surgical procedures, permanent, root resorption

## Abstract

**Background:**

External cervical root resorption (ECRR) is an uncommon and often asymptomatic condition whose early diagnosis is challenging. When located on the buccal aspect of posterior teeth, treatment should address both lesion control and long‐term periodontal stability.

**Case Presentation:**

A 67‐year‐old healthy, nonsmoking female presented with RT1 gingival recessions in the mandibular right sextant and a cervical defect on Tooth #28. Clinical and radiographic evaluation confirmed ECRR with a favorable prognosis. Treatment included a partial‐thickness flap for surgical access, complete debridement of the defect, minimal osteoplasty, and restoration under rubber dam isolation. A free gingival graft harvested from the palate was placed to increase keratinized tissue and deepen the vestibule. At 3‐ and 15‐month follow‐ups, the tooth remained vital and asymptomatic, the graft showed stable integration, and radiographs demonstrated no progression of the lesion.

**Conclusions:**

In non‐esthetic posterior areas, a combined restorative–periodontal approach with a free gingival graft can successfully manage ECRR while enhancing keratinized tissue and supporting long‐term periodontal health.

Summary

The free gingival graft (FGG) is an effective surgical solution for external root resorption in non‐aesthetic areas, enhancing the quality and quantity of keratinized tissue.

## 1. Introduction

External cervical root resorption (ECRR) is considered a form of external resorption of inflammatory origin and represents a challenging clinical condition in terms of diagnosis and prognosis [1–3]. It is characterized by progressive loss of calcified tissues—dentin, root cementum, and alveolar bone—at the cervical region of the root. In early stages, pulpal physiology is usually preserved, and the lesion may remain undetected on routine clinical and radiographic examination [2–7]. The etiology of ECRR is often unclear, and multiple predisposing factors have been proposed, including trauma, orthodontic tooth movement, and internal bleaching [1, 2, 6–10].

Because the causal factor is frequently unknown, prevention is difficult and treatment is strictly dependent on the location, extent, and accessibility of the lesion. Key elements for a favorable prognosis include early diagnosis, meticulous removal of resorptive tissue, appropriate restorative and, when needed, endodontic management, and surgical treatment of the surrounding periodontal tissues [1]. Most reports describe ECRR in anterior teeth, where esthetic demands prioritize surgical approaches using connective tissue grafts [2, 11–13]. In contrast, when ECRR occurs in non‐esthetic posterior sites, treatment can be tailored to optimize access, maintainability, and soft‐tissue stability rather than complete root coverage. This case report describes a combined restorative‐periodontal approach using a FGG to manage ECRR in a mandibular premolar, with the dual objective of controlling the lesion and enhancing the quality and quantity of keratinized tissue for long‐term periodontal health.

## 2. Case Presentation

A 67‐year‐old female in good systemic health and with no known allergies was referred to the Periodontics Department for management of gingival recessions in the mandibular right sextant. Her chief concern was the progressive apical migration of the gingival margin and increasing sensitivity. The patient was a nonsmoker, reported no parafunctional habits, and she had never undergone orthodontic or bleaching treatments.

### 2.1. Clinical Examination

Intraoral examination revealed RT1 gingival recessions [14] affecting Teeth #26, #27, and #28 (mandibular right canine and premolars). The area exhibited reduced keratinized tissue, a shallow vestibule, and mild dentin hypersensitivity. Probing depths ranged from 1–3 mm with no bleeding on probing. Tooth vitality tests were positive.

On the buccal aspect of the mandibular right first premolar (Tooth #28) a localized cervical defect was noted. Although differential diagnosis, such as carious and noncarious cervical lesions, was properly evaluated, through the X‐rays and the clinical exploration with a probe revealed a hard, irregular resorptive surface, suggestive of ECRR.

### 2.2. Radiographic Assessment

A periapical radiograph confirmed a superficial radiolucency at the cervical third of Tooth #28, consistent with ECRR. The lesion was confined to the coronal third, with no pulpal involvement or periapical pathology. The tooth was therefore classified as having external cervical resorption with a favorable prognosis according to Heithersay′s criteria [2].

### 2.3. Diagnosis and Treatment Plan

The clinical findings supported a diagnosis of ECRR at Tooth #28, classified as Heithersay Class 2 [2], together with RT1 [14] gingival recessions affecting Teeth #26–28, reduced keratinized tissue width, a shallow vestibule, and dentin hypersensitivity. Considering the buccal location of the resorptive lesion and the patient′s discomfort during oral hygiene, a combined periodontal–restorative approach was planned. Surgical access was achieved through a partial‐thickness flap extending from the canine to the second premolar, allowing full exposure and thorough debridement of the resorptive defect. Minor osteoplasty was performed on the buccal plate to regularize the bony contours and facilitate restoration. The defect was then sealed under rubber dam isolation using composite resin. To enhance soft‐tissue quality and long‐term stability in this non‐esthetic posterior region, a FGG was placed to increase the band of keratinized tissue and deepen the vestibule. This comprehensive plan is aimed at arresting the resorptive process, improving periodontal stability, and supporting long‐term plaque control.

### 2.4. Surgical Phase

**Figure 1:**
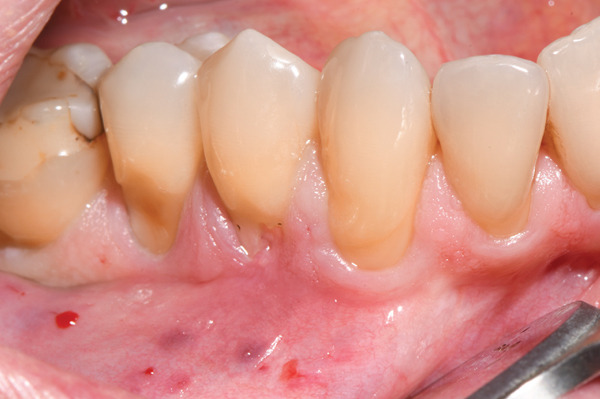
Preoperative clinical view of the mandibular right lateral sextant, after local anesthesia.

The patient provided both oral and written informed consent for treatment. Local anesthesia was administered (2% xylocaine with 1:100,000 epinephrine) (Figure 1). The incision is performed at the mucogingival line, starting distally, from the second premolar to the canine; a 45° beveled “hockey stick” incision is carried out mesial to the canine.

**Figure 2:**
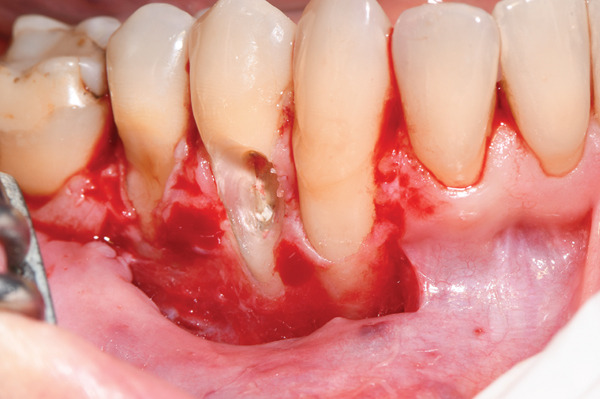
Bleeding periosteal recipient bed is prepared, and the complete removal of the infiltrated cementum and dentin and the exposure of the pulp‐canal cavity are performed.

As planned, a partial thickness flap was elevated, preserving papillae (Figure 2). The removal of infiltrated cementum and soft dentin by resorption tissue is performed with a small rounded floated tungsten carbide burs and medium‐grain diamond cylindrical burs mounted on a “red ring” high‐speed handpiece. Minimal osteoplasty was performed to regularize the bone contour and facilitate access for restoration. Hemostasis was achieved, and the site was isolated with a rubber dam.

The cervical defect was restored using composite resin, ensuring smooth margins and proper integration with the root surface.

**Figure 3:**
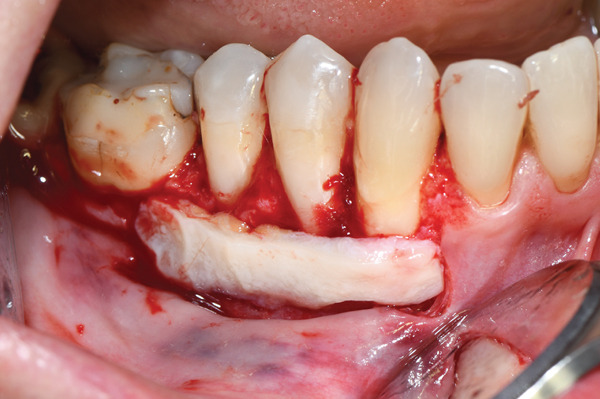
The free gingival graft harvested from the palate was immediately positioned on the recipient site.

A FGG of approximately 2 mm‐thickness was harvested from the palate and sutured to the recipient bleeding bed, apically to the previously exposed root surfaces (Figure 3). The donor site was sutured with horizontal compressive mattress stitches, crossed in the palate, and knotted buccally.

**Figure 4:**
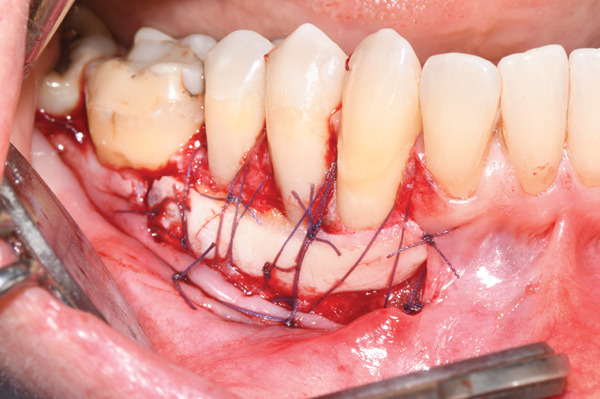
The epithelial‐connective tissue graft was sutured with resorbable sutures, and the alveolar mucosa stabilized apically to the underlying periosteum.

The graft was secured to the recipient site using a horizontal periosteal mattress suture placed apically to ensure intimate adaptation to the underlying vascularized periosteal bed. One stabilizing suture was placed for each treated tooth. The buccal mucosal flap was then repositioned and sutured apically to the graft using simple periosteal stitches (Figure 4). This approach enables simultaneous augmentation of the keratinized tissue band and improvement of vestibular depth.

### 2.5. Postsurgical Protocol

Postoperative discomfort was managed with ibuprofen 600 mg twice daily for 2 days. The patient was instructed to avoid mechanical trauma and to refrain from toothbrushing in the surgical area for the first 2 weeks. Chemical plaque control was maintained with a 0.12% chlorhexidine mouthrinse used three times daily for 1 min. Palatal sutures were removed after 7 days, whereas the sutures at the recipient site were removed at 14 days postoperatively.

**Figure 5:**
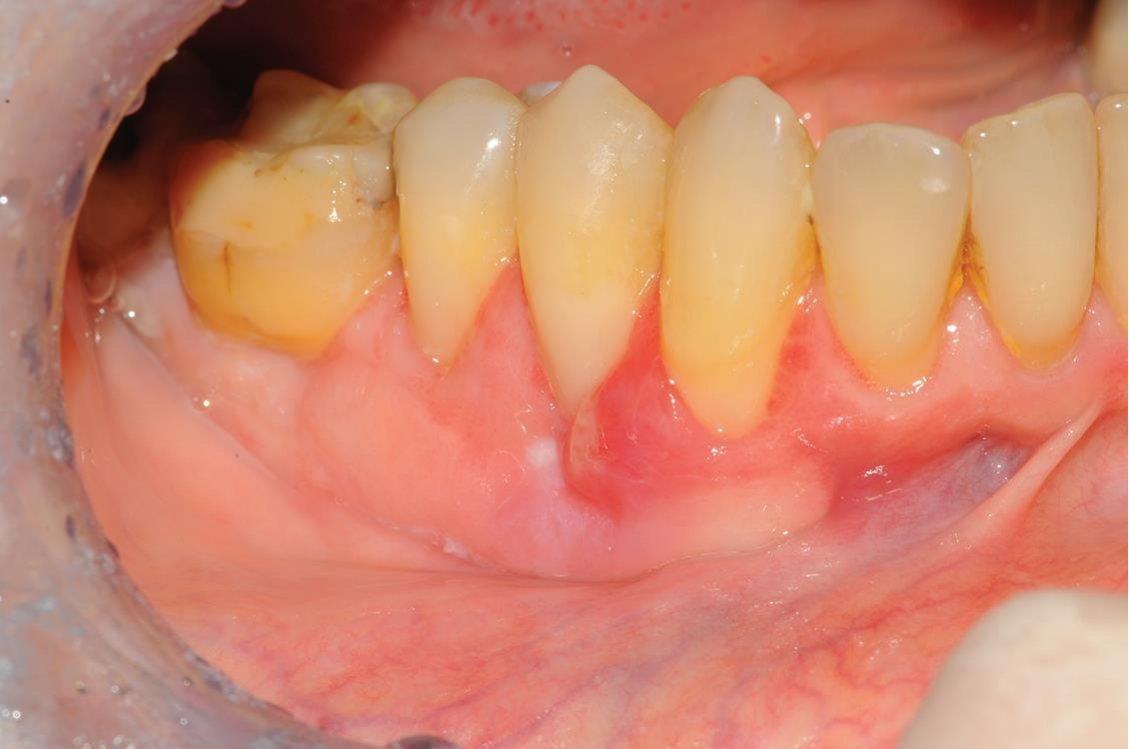
Clinical aspect after 2 weeks postsurgical on the vestibular mandibular lateral sextant.

**Figure 6:**
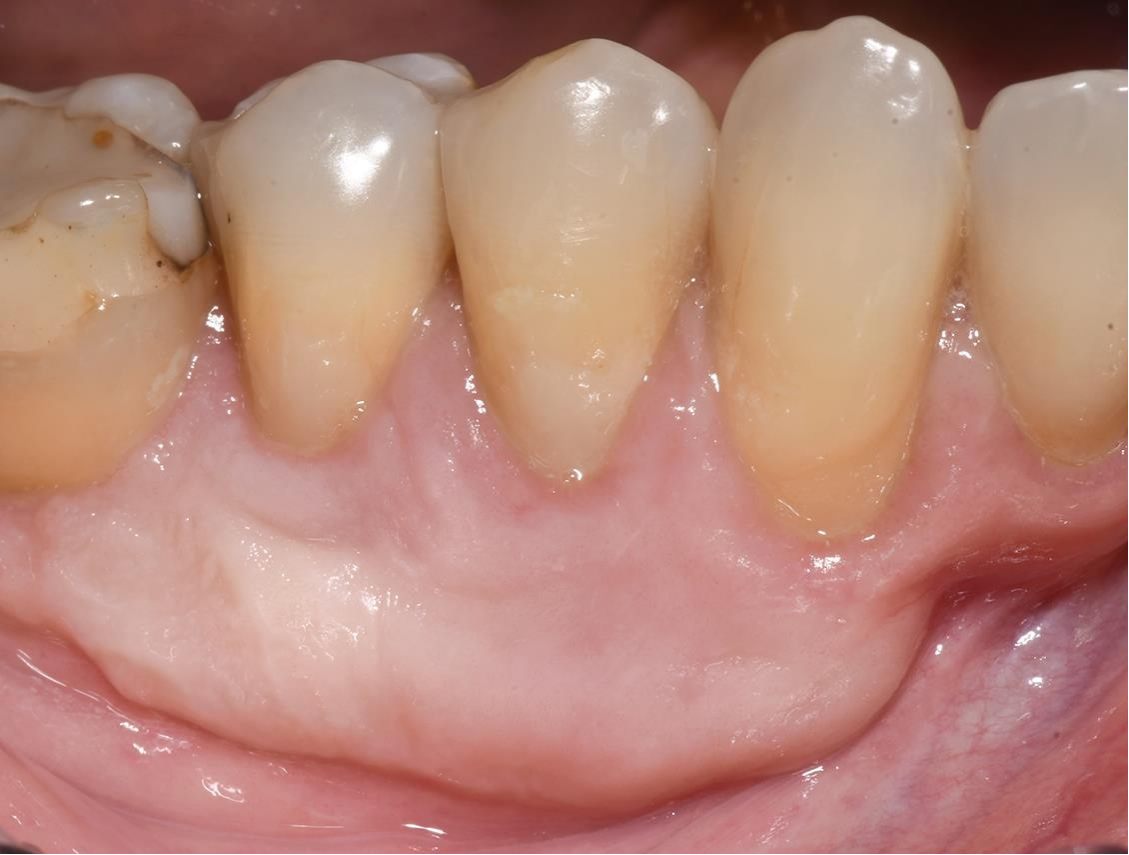
A 15‐month follow‐up.

Postoperative symptoms were within normal limits, and initial healing progressed uneventfully. Follow‐up appointments, including professional supragingival cleaning and individualized oral hygiene reinforcement, were scheduled at 1, 3, 6, and 12 months. Healing maturation and soft‐tissue stability were assessed at each visit (Figures 5 and 6).

## 3. Discussion

ECRR represents a challenging clinical condition due to its silent progression, complex etiology, and the potential for diagnostic delay. In most cases, the lesion originates in the cervical region and may be associated with developmental disturbances or mechanical and chemical trauma [2, 5]. Among predisposing factors, orthodontic tooth movement is consistently recognized as a major contributor, likely due to pressure‐induced alterations in the protective cementum layer [5, 8]. In the present case, the patient reported no history of trauma but did describe extensive orthodontic therapy during adolescence, which may have compromised the integrity of the cervical cementum and facilitated direct exposure of dentin to resorptive periodontal cells [9, 15].

Understanding the causal background is essential not only for establishing prognosis but also for determining the timing and need for endodontic therapy. As highlighted by Gonzales et al., assessing potential trauma or iatrogenic factors guides the clinician in determining whether pulp involvement is imminent [15]

Similarly, Yoshpe et al. documented recurrent cervical resorption in a patient with previous orthodontic treatment, emphasizing the long‐term vulnerability of teeth with altered cervical cementum [16].

A critical aspect of management is the decision regarding endodontic treatment. In this case, root canal therapy was deferred until surgical access permitted direct evaluation of the defect and pulpal status, as the tooth remained vital and only mildly sensitive. This contrasts with the approach described by Gonzales and Rodekirchen [15], who performed endodontic treatment preoperatively despite signs suggesting that the pulp was not yet infected. Current literature indicates that endodontic therapy should only be initiated when pulpal involvement is confirmed or strongly suspected [17], and a recent systematic review supports its selective use for lesions at risk of rapid progression [18]. In agreement with these findings, the present case reinforces that pulp vitality can often be maintained when the lesion is limited to the cervical region and adequate debridement and sealing can be achieved.

The surgical approach remains a key determinant of long‐term success. Most published case reports describe treatment of ECRR in anterior teeth, where esthetic outcomes drive clinicians to favor connective tissue grafts [19–21]. In contrast, when lesions are located in non‐esthetic posterior segments, treatment priorities shift toward access, maintainability, and periodontal stability. In this case, a FGG was deliberately chosen instead of a connective tissue graft to optimize plaque control and reinforce the band of keratinized tissue. Leaving a portion of the composite‐restored root surface intentionally exposed enhances cleanability and reduces the risk of microleakage at the restoration–tissue interface. At the same time, the increased keratinized tissue and re‐established vestibular depth support long‐term periodontal health and protect adjacent sites from further recession. This approach also resolved the patient′s dentinal hypersensitivity through direct intraoperative restoration.

Overall, the clinical protocol adopted in this report emphasizes conservative tooth retention when feasible, direct evaluation of pulpal integrity during surgery, and the use of soft‐tissue augmentation tailored to the functional demands of posterior regions. The successful healing and 15‐month stability observed in this case underscore the value of combining meticulous surgical debridement, appropriate restorative sealing, and a strategically selected mucogingival technique to achieve both biologic and functional success.

## 4. Conclusions

Intraoperative access provided by the surgical flap allowed for precise evaluation and complete debridement of the cervical resorptive defect, enabling a more accurate assessment of tooth prognosis. Restorative sealing performed under controlled isolation ensured optimal marginal adaptation and facilitated long‐term stability of the repair. In non‐esthetic posterior regions, intentionally maintaining the composite restoration outside the gingival sulcus promotes maintainability by improving plaque control and minimizing the risk of microleakage.

The decision to combine defect debridement, restorative management, and soft‐tissue augmentation with a FGG supported both functional and periodontal objectives. This approach not only preserved pulpal vitality but also increased the width of keratinized tissue and improved vestibular depth, contributing to favorable long‐term periodontal stability. Overall, this case demonstrates that a tailored restorative–periodontal protocol represents a predictable and conservative treatment option for managing ECRR in posterior sites where esthetics are not a primary concern.

## Funding

No funding was received for this manuscript.

## Consent

Written informed consent was obtained from the patient for publication of this case report and any accompanying images.

## Conflicts of Interest

The author declares no conflicts of interest.

## General Statement

*Clinical significance*. External cervical root resorption can present diagnostic and therapeutic challenges, particularly when detected late or located on posterior teeth. This case illustrates that, when pulpal vitality is preserved and surgical access is feasible, a conservative combined restorative–periodontal approach can effectively manage the defect while supporting long‐term periodontal stability. The use of a free gingival graft in a non‐esthetic posterior area enhances the band of keratinized tissue, improves vestibular depth, and facilitates plaque control—key factors in preventing further attachment loss and recurrence. This protocol offers clinicians a predictable and maintainable treatment option for posterior ECRR, emphasizing functional success over esthetic coverage.

## Data Availability

The data that support the findings of this study are available from the corresponding author upon reasonable request.
